# East and West

**Published:** 1887-10-22

**Authors:** Leslie Keith

**Affiliations:** Author of "The Chilcotes," "Uncle Bob's Niece," etc.


### East and West.

By Leslie Keith,

Author of "The Chilcotes," "Uncle Bob's Niece," etc.

*(Continued from p. 49.)*

### Chapter. IV.—Poor Relations.

The year was but young when the two wanderers from the North came up to London, and July was drawing to a close when Alicia Frankland came downstairs one morning, to find care seated on her father's brow. That amiable gentleman so seldom looked anything but what might be expected of a papa who was blessed with such exceptional daughters, that Alicia was arrested by the unusual gravity of his expression. She came in trailing a very wonderful confection—isn't that the recognised word?—over the carpet, but midway between the door and the table she paused.

Mr. Frankland's ear catching the cessation of her draperies' rustle, he looked up, and his brows lifted themselves whimsically. There was a dawning smile in his eyes—a humorous smile, as if some contrast stirred him to a faint amusement.

"Papa," said Alicia, her glance wandering to a large sheet of blue paper he held in his hand, "if that is a lawyer's letter, or a tax-paper, or a policy, or anything equally disagreeable, I beg you will put it out of sight."

"It appears," he said gravely, "to be something between an appeal and a menace."

"An appeal—from whom?"

"I have not quite succeeded in making out the details," he said modestly, "but so far as I can gather, it appears to be the history of a certain David Jardine's 'forbears'—that is the term, I believe." He put up his gold-rimmed glasses and sought out the offending sentence.

"'Forbears' means ancestors," said Alicia the well-informed, then she went on: "Is it the ancestors who threaten? It is a new attitude for ghosts; it might be instructive to the psychical-research people."

"Perhaps it would amuse you to look over it?" Mr. Frankland tendered her the paper, as he lay back in his chair with a sigh. "It may prepare you for a visitor."

"I don't like visitors," said Alicia, calmly; "they give trouble, and are seldom amusing; but a ghost as a guest might be less uninteresting."

"Papa!" cried Kitty, in alarm, "it isn't a ghost!"

"It is a young man named David Jardine, who claims to be your cousin. I think you will find him very substantial indeed—very—corporeal—very Arcadian." He groped about fruitlessly for a better word. "He is a shepherd and small farmer in the North."

Alicia looked at him majestically with her chin in the air. Then she turned away.

"Give me some coffee, Kitty, please," she said. It was evident from her demeanour that she recognised in her father's words one of those jests that are dangerous to family peace. When Alicia put on that air it was understood that the subject had passed beyond the region of discussion, and the meal proceeded in a comfortable silence that was very soothing to the appetite.

When Mr. Frankland had finished—and he was a good if a discriminating trencherman—Alicia rose and appropriated the blue paper, which was none other than that laborious document drawn up by the schoolmaster for the confounding of the Frankland pride.

"I will examine it, since you wish it," she said. "If it is an authentic ghost-story—"

"It is the story of another lost illusion, I'm afraid. Take it, my dear, and make what you will of it. If it bores you—"

"If it bores me I shall not spend any time over it. There is always the comfort of being able to try something else."

Her preparations for the examination of the document were elaborate. She shut herself up in her own room and told her maid that she was not to be disturbed. She was serious as becomes a modern scientist who prepares himself to interview a possible spook. It was something to do—something to fill up the morning's tedium; perhaps—oh, flattering hope!—it might be something new and interesting.

In the absence of her sister, Kitty's tongue was loosed.

"Papa"—she pursued her father into the smoking-room—"is your 'lost illusion' truly a relation? Is it true that we have a cousin—a cousin who is a common person—a farmer?"

"So it would seem. Two cousins, indeed."

"Two! what shall we do?"

"I shall try to bear up under the dispensation," he said, so gravely, that she laughed.

"How will Alicia take it?" she questioned. "Will she find it amusing, or—will she annihilate you? Oh, my poor, dear papa, I'm afraid there is a very painful quarter-of-an-hour in store for you!"

Alicia "took it" at least with deliberation. The blue document was not sent flying downstairs in the hand of a trembling page: there was hope in that. Kitty listened outside her sister's door, but her strained ear met nothing but a baffling silence. Alicia might have fallen asleep over the Jardine pedigree; the study of a pedigree frequently has this effect when it does not madden or drive to despair. She did not rejoin her family till the lunch-bell had sounded, and then she came down, wearing her riding-habit.

"Papa"—she walked straight up to him—"here is your paper. I have given it my best consideration; I do not think it will add either to our comfort or our amusement to know these people—they are at best cousins in the second or third degree, and that tie ought never to be recognised unless the person in question happens to be a duke, or a marquis at the least. I should consider it a duty in such a case to remember the connection"—she spoke with a cynicism that did not lack entire frankness.

"You would remember it, I don't doubt, 'without the help of the herald.' We are, then, to ignore our new-found relations?"

"Let us ask them to dinner," suggested Kitty; adding, in bold parody of her sister's manner, "it might possibly be entertaining after all."

"I think," Alicia went on, deaf to this interruption, "a cheque would meet the case."

"You forget—the young man is a poet as well as a farmer. Poets are sensitive; it might hurt his feelings."

"I do not see why that should hurt mine," said Alicia, judicially. "I am not a sensitive person where others are concerned. It might annoy me to have him to dinner, as Kitty proposes, because he would certainly not know how to use his knife and fork; he would take the wrong wines and mix his sauces, and that would be disagreeable to witness; but if he were foolish enough to return your money, that would not distress either of us. We should be supported by the conviction that we had done all that was required of us, and you would probably spend the cheque on cigars."

"It is certainly not a troublesome way out of the difficulty."

"It is an easy way—a way for which you ought to be grateful. You ought to be humbly thankful to Kitty and me for letting you off so lightly. It was really very inconsiderate of you to allow people of that kind to enter the family."

"I fail to see how I am responsible," said Mr. Frankland, bearing up under this accusation with fortitude.

"You ought to have thought of *us*," Alicia went on rebukefully. "You ought to have advised your cousin not to marry a person who would bring shepherds and farmers into the family. You had better come and ride with me now. I scarcely care to trust you out of my sight."

When the horses were brought to the door and the riders stood on the steps, Alicia, critically surveying her perfectly groomed mare, was startled by a sudden whining voice in her ear, and turning, saw that a beggar had crawled up the steps and was making the conventional appeal for charity, no policeman being within sight. Alicia looked at her with a curious, half-fascinated gaze—a woman, this, like herself, but a woman in tatters and in want. She wheeled round to her father.

"Give me some money," she said.

"Mr. Frankland looked from his daughter to the bundle of ragged aches and ails at her side.

"Though I know that you are too philosophic to be disturbed by her," he said, "John may as well send her away."

"Give me some money," Alicia repeated urgently. She took the silver he deliberately extracted from his pocket, and dropped it into the clutching fingers. The beggar went trailing her way down the steps, dealing out blessings on the generous lady in a voice that was quite as apt at maledictions when the lady was not generous.

"My florin will go to enrich the nearest publican," said Mr. Frankland, watching her departure with half-shut eyes. "That is a comforting thought."

"What do I care!" cried Alicia, turning on him with unexpected fierceness. "I don't feel sorry for her; I don't pity her; I don't care whether she starves, or dies of drink! It is myself I am sorry for. She came too near me; it—it was too unpleasant a contrast. That was why I gave her money, to make her go away."

He had never seen her so moved. She was in a sombre mood for all the day, which happened to be an idle one in Mayfair.

In the evening she held a little reception; one of those unconventional gatherings in which Mr. Frankland was supposed to take peculiar delight, though he manifested it by modestly remaining in a corner. "Papa's Thursdays," his girls called them, but possibly—just possibly, the men who obediently quitted their clubs and came to Park Lane with admirable punctuality would not have had so conscientious a memory for Mr. Frankland's Thursdays had he not been the father of two most desirable daughters.

As usual, the stream of worshippers divided: the younger, headed and led by Jack Fane, surrounding Kitty; the older and more staid, who love a piquant tongue as well as a pretty face, bringing their devotion to Alicia.

Alicia had two problems for her admirers to solve that night, which she presented to each in turn.

"What is a farmer?" was the first of them; "and what is a poet?" was the second. "I have looked in the dictionary," she said, "and I find that a farmer is a cultivator of leased ground; and a poet is 'the author of a poem.' The last definition is particularly convincing. It has struck me that your joint wisdom might help me."

"But a farmer, you know," said one of them with mild remonstrance. "Why, you see, there are all sorts of them. Lord A. is a farmer, and Sir B., and Captain C., and so is the poor devil down in the shires who can't pay his tithes or put bread in his children's mouths."

"My farmer is of the latter order," said Alicia, calmly. "He is so poor that he has no acres under cultivation at all. You do not meet such at your clubs! You would not go out of your way to elect a rough young man from the country who had failed to keep the few acres his father left him, even if he were a poet?"

"A poet, you see," said another; "the word's as vague as farmer. There's Tennyson and Browning, you know, and the other nobs; and there's the fellow who writes to crack up somebody's blacking, or soap, or boots."

"Oh," said Alicia, "you call him a poet also? What a very accommodating term this is!"

At night she summed up all the opinions she had heard, and reviewed them judicially. A farmer of the lesser order was a person who wore hob-nailed boots, and was red and rough—a frequenter of ale-houses and a lover of a broad jest on market days; a whistler in his vacant hours, and at his busier, with no thoughts that could rise above his crops and his beasts. Clearly, neither as a tiller of the soil, nor as the celebrater in rhyme of Brown's soap, or Green's blacking, or White's cocoa, could such an one feel at home in the gracious society of Mayfair or be happy there. It would be but a cruel kindness to invite him to breathe that rarer air, to mount to heights that were not meant for such as he.

"I was right, I was right," she said to herself as she paced the room, "I was right to spare him. And yet it need not have troubled me much if he had suffered. If a man will be a fool, he must take the risks of his folly."

The season was now very near an end, at its last gasp, indeed, and with the waning days Alicia took her pleasures more strenuously and with less of fastidiousness, as one who fears a coming time when there shall be no weapon at hand to ward off tedium. Some one, influenced by the noble example of Lady Ashton, had invented a new diversion in the shape of a whistling party, at which lady *siffleurs* were to have opportunity to distinguish themselves. It was a pretty conceit, and though a strict propriety has always frowned on this accomplishment when girls furtively practise it, there is no crime in laughing lips tuning themselves to this kind of music. Alicia, however, who had come off with so much distinction at the soap-bubbling, declined to lend her countenance to the new play. She had tried the art once before her glass, but her lips refused to sanction this foolery. She frowned at herself in dark disgust.

"Am I beginning to fail—to yield?" she asked herself with a note of alarm. "I must not; I shall not," she menaced herself. What did she fear? Was it a sick distaste of the life she had elected to live—a dread lest her cynicism should fail her? It is but hazarding a guess, and yet it might be so.

In a day or two she asked her father if he had sent the cheque.

"I sent it."

Her eyes asked something more, and he answered their appeal.

"You gave me permission to buy cigars with it—under certain circumstances." He significantly held out the one he had taken from his lips when she entered. "You want to know more? Well, he rejected it, after the fashion of—a poet. No, my dear, I did not keep the letter. I believe it went in the same service as the cheque—to light my pipe."

That day after lunch, Kitty, who had silently drunk in the accents of this dialogue, came to her sister wearing a little air of determination that seemed an odd parody of the elder's habitual mood.

"If you don't want the carriage for an hour or two, Alicia, I should like it this afternoon."

"I shall ride. You are going shopping?"

"I am going to a place that is called Bethnal Green, to visit two people who call themselves my cousins. You and papa ignore them without having seen them. I mean to see them first."

Did this bold scheme cross some secret plan of Alicia's, that she looked upon it with such cold disfavour? Perhaps it had been her intention to ride to this same unknown land of the East, but assuredly her horse's head should not now be set that way.

"Very well," she said, with chilling indifference. "As it pleases you; but to be consistent you ought to go in an omnibus."

"No—" Kitty shook her sunny head. "Omnibuses are traps and snares for the unwary—so I've heard. Nasty little germs and catching things lurk there. On the top it is different; there you are liable to nothing more than smuts and the odour of bad tobacco—so Jack tells me; but these delights are not for me."

"Jack?"

"I will allow him to go with me," said Kitty, meekly, "but not into the house. He must be broken gradually to the sad fact that we have pauper cousins. He didn't know it when he asked me to marry him, poor Jack!"

Alicia did not ride that day, for some reason that it was her sovereign will not to disclose. If she kept instead a furtive watch for the return of the travellers, they had not the satisfaction of knowing it. She was seated in a corner of the drawing-room most remote from the windows, apparently wholly absorbed in the persual of a book, when Kitty burst in there.

Kitty was too full of her tidings to deal them out sparingly. She hurled them forth with a prodigal hand.

"Oh, Alicia! what do you think! You will never guess!" she cried.

"Probably not, since I shall not try," said Alicia, indifferently.

"Well, even you will be surprised. Papa has been to see them—twice!"

(*To be continued.*)

